# User‐Centered Evaluation of Healthcare Information System Utilization: A Cross‐Sectional Study Integrating Acceptance Models

**DOI:** 10.1002/hsr2.72786

**Published:** 2026-08-26

**Authors:** Fatemeh Aghilinasab, Mozhgan Tanhapour, Mohamad Jebraeily, Bahlol Rahimi

**Affiliations:** ^1^ Health and Biomedical Informatics Research Center Urmia University of Medical Sciences Urmia Iran; ^2^ Department of Medical Informatics School of Allied Medical Sciences Urmia University of Medical Sciences Urmia Iran; ^3^ Department of Health Information Technology School of Allied Medical Sciences Urmia University of Medical Sciences Urmia Iran

**Keywords:** hospital information system, technology adoption, user need, user satisfaction

## Abstract

**Background and Aims:**

Despite the promising role of health information systems (HISs) in healthcare settings, they have not consistently met users' expectations. The current research aimed to investigate the extent to which the functions of a healthcare information system align with users' actual needs, while also identifying the factors that influence the system's utilization.

**Methods:**

The study was conducted in 2021 at a tertiary university hospital in Urmia, Iran. It utilized a quantitative method with a descriptive, cross‐sectional design. Data were collected using a researcher‐made questionnaire from 32 administrative and clinical users of the hospital information system, employing a stratified sampling approach. We used SPSS version 27 for data analysis.

**Results:**

Among the participants, paramedical staff have the highest level of satisfaction (mean difference = −0.42, 95% CI [−0.82, −0.02]) and system utilization (mean difference = −0.25, 95% CI [−0.46, −0.05]). Exploratory subgroup comparisons suggested potential differences in “familiarity with IT” across age groups (mean difference = 1.04, 95% CI [0.63, 1.46]) and work experience groups (mean difference = 0.53, 95% CI [0.06, 1.00]). The “user satisfaction” has a positive correlation with “system utilization” (*r* = 0.830, *p*‐value < 0.001), “matching the system with users' needs” (*r* = 0.626, *p*‐value < 0.001), and “training and supporting” (*r* = 0.597, *p*‐value < 0.001) variables. “Matching the system with users' needs” is also positively correlated to “system utilization” (*r* = 0.713, *p*‐value < 0.001).

**Conclusion:**

This study is expected to improve HIS utilization, healthcare quality and productivity, and healthcare organizations' financial returns. Our findings have insightful implications for those interested in the successful implementation of HIS systems, including hospital managers, chief executive officers, health information technology personnel, and HIS vendors.

**Abbreviations**
DOI: diffusion of innovations
HIS: health information system
IT: information technology
TAM: technology acceptance model
UTAUT: theory of acceptance and use of technology unified

## 1 Introduction

The management of healthcare organizations in the contemporary era is impossible without information technology [1]. Hospital information systems (HISs) are a type of health information system focused on hospital operations [2]. They play a fundamental role in healthcare by enabling modern practices and their associated benefits [3]. However, their effectiveness is not determined solely by technical implementation, but also by how well they are accepted and used by healthcare professionals [4].

Despite the HISs potential, its implementation outcomes remain inconsistent. Studies show that in low‐ and middle‐income countries (LMICs), only 28% of HISs have met investors' expectations and performed successfully [5, 6]. These challenges are not limited to LMICs. Studies from high‐income countries report persistent usability issues and moderate user satisfaction, with only a minority of clinicians reporting positive experiences and many questioning their impact on efficiency and patient safety [7, 8, 9, 10]. This highlights a gap between system deployment and actual realized value in practice, particularly in relation to human and organizational factors.

One of the most critical dimensions for complex information systems, such as HISs, is user and technology adoption [11]. System acceptance is critical, as benefits can only be realized when intended users accept and actively use the system [12, 13]. Accordingly, understanding user behavior has become a central concern in evaluating health information systems [14].

Several studies on the acceptance and use of information systems in healthcare have emphasized the users' behavioral intention [15, 16]. Nevertheless, approximately 30% of HIS projects have failed to meet the expectations of healthcare decision‐makers and policymakers [17, 18, 19]. Such shortcomings have contributed to healthcare professionals' frustration and resistance to using these systems [20]. This underscores the importance of assessing users' attitudes, which influence both behavioral intention and actual system utilization [21, 22]. Factors such as age, educational level, time spent working with the system, and system usage experience can affect the user's attitude and, consequently, the user's interaction with the system [23].

However, in LMICs, hospitals often implement pre‐purchased HISs. Therefore, most users deal with mandated systems, and their system usage is driven by external pressures, such as managerial or institutional mandates, rather than intrinsic motivation. Given these conditions in LMICs, using integrated evaluation methods to measure both users' mental and practical behavior seems more reliable for understanding system acceptance and usage [24].

**Figure 1:**
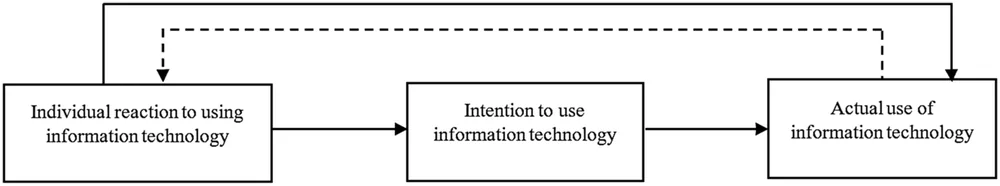
Main factors in user technology acceptance models.

There are several approaches, including frameworks, models, and tools, for evaluating health information systems with a focus on end‐user technology adoption, such as the technology acceptance model [25] and the unified theory of acceptance and use of technology (UTAUT) [26]. Each has unique characteristics [27, 28, 29]. The core concept of them is presented in Figure 1. While these models provide valuable insights, they have limitations. They rarely consider the influence of user attitude in contexts where system use is mandated rather than voluntary [30, 31]. Moreover, there is no consensus on a single model that fully explains HIS adoption, particularly in LMICs [15, 16, 17, 32].

To address these limitations, this study takes an integrated approach by combining existing frameworks and explicitly incorporating user attitude as a key determinant of behavioral intention. Because HIS utilization depends on aligning system features with users' needs and understanding the factors that shape system use, this study investigates the associations between users' attitudes, training, satisfaction, and technology adoption in the context of an HIS at a tertiary university hospital in Iran.

## 2 Methods

We investigate the relationships among users' attitudes, training, satisfaction, and technology adoption using a quantitative, descriptive, and cross‐sectional approach [33]. We selected this methodology to systematically analyze key aspects and assess their relationships in an actual work environment. The study's context, data gathering processes, sampling method, and statistical analysis are detailed in the following subsections.

The ethics committee of Urmia University of Medical Sciences approved this study (IR.UMSU.REC.1398.104). All procedures were carried out in accordance with the regulations and standards set forth by the Urmia University of Medical Sciences Ethical Committee. Participants were asked to complete an informed consent form before they participated in the study.

### 2.1 Study Setting

The study was conducted in 2021 at the Seyyed‐ol‐Shohada University Hospital in Urmia, Iran. The hospital had more than a decade of experience with hospital information systems.

### 2.2 Data Collection

The development of the questionnaire began in early August 2020. We attempt to combine the existing frameworks by considering the user's attitude as a factor influencing behavioral intention. Initially, the evaluation types and results of the existing information systems in Iran were examined. Subsequently, relevant documents were studied, and interviews were conducted with information system vendors and technical experts of the studied hospital. The most pertinent questions regarding the research environment and purpose were selected and classified using well‐established frameworks, such as TAM [25] and UTAUT [26], as these are valid and reliable measures of user satisfaction with a system [34]. The resulting questionnaire consists of six sections, including demographic information, familiarity with IT (questions about working with technology), training and support (questions about support from the managers and getting enough education to work with the system), user satisfaction (questions about feeling comfortable the user when working the system), using the system (question about the degree of ease in managing tasks while using the system), and matching system with users' needs (questions about the compatibility of using the system, flexibility of the system to user's needs, and participatory design). The questionnaire included a 5‐point Likert scale, ranging from “agree” (scored 5) to “strongly disagree” (scored 1), with “no opinion” scored 3. It is worth noting that the validity of the research tool was verified and approved by five experts in medical informatics and health information management, as well as a senior technical expert. The reliability of the questionnaire was approved through the random selection of 10 participants and the Cronbach's alpha test. The Cronbach's alpha value for all parts was higher than 0.7, indicating acceptable reliability [35].

### 2.3 Sampling Procedure

The administrative and clinical HIS users were recruited using a stratified sampling approach. The inclusion criteria were as follows:

- Employed as an active end‐user (administrative or clinical staff) of the HIS at Seyyed‐ol‐Shohada University Hospital, Urmia, Iran, during the data‐collection period (January–December 2021).
- Had used the HIS for at least 6 months at the time of questionnaire distribution (to ensure sufficient familiarity with the system).
- Gave their signed agreement to participate in the study.

Also, exclusion criteria were as follows:

- Hospital managers, physicians, or medical residents (they already expressed their unwillingness to participate in the study, therefore systematically unavailable during the study period).
- The temporary, trainee, or extern staff with less than 6 months of experience using HIS.
- Returned questionnaires with more than 20% missing responses, marked as “no opinion” across entire sections. For the remaining questionnaires, analyses were performed using only available data for each item, with no imputation of missing responses.

The hospital information system of the target hospital had 35 active end users. Based on Cochran's formula [36] (with an alpha level of 0.05), a sample size of 32 individuals was deemed sufficient for the analysis. Therefore, 35 questionnaires were printed and distributed among end users from January to December 2021. Because our population comprises different subgroups, stratified sampling ensures that each subgroup has a chance to be represented, which is important for the generalizability of our results [37]. Based on the stratified sampling approach and the population of each part of the HIS system in the target hospital, the number of participants in each group is determined in Table 1.

**Table 1:** Number of participants in each hospital section.

Users group	Radiology	Secretory	Laboratory	Medical record	Financial section	Pharmacy	Nurse
Number of active users	1	2	3	4	3	2	19
Sample size	0.9	1.8	2.7	3.7	2.7	1.8	17.4
Rounded sample size	1	2	3	4	3	2	17
Total sample size	32						

### 2.4 Statistical Analysis

Participants' characteristics and responses were primarily analyzed using descriptive statistics, including frequencies, percentages, means, median, interquartile range, and standard deviations.

Given the relatively small sample size (*n* = 32) and heterogeneity of respondents' professional backgrounds, inferential statistical analyses were conducted cautiously and are considered exploratory. To improve subgroup comparability, selected variables (e.g., age, job position, work experience, and educational level) were regrouped into broader categories.

For comparisons between two groups, independent samples *t*‐tests were applied when assumptions were reasonably met [38]. No formal hypothesis testing framework was applied; *p*‐values are reported descriptively to indicate patterns in the data. For variables with small subgroup sizes (e.g., educational level and daily working time duration), non‐parametric alternatives, such as the Mann–Whitney exact test and Kruskal–Wallis were used.

The strength and direction of the relationships between the research variables were analyzed using the Pearson correlation coefficient. To determine participants' overall perceptions of the research variables, a one‐sample *t*‐test was performed to compare the variables' average scores with the expected value of 3.

All tests had two‐sided *p*‐values, and *p*‐values less than 0.05 were considered statistically significant. Also, results with *p* < 0.01 were highly significant [38]. Estimates of effects are given as differences between groups' means, Cohen's *d*, or differences between the mean and the test value, along with 95% confidence intervals (CI) and *p*‐values.

All statistical analyses were pre‐specified. Due to the limited sample size and unequal group distributions, results of inferential tests should be interpreted with caution and are presented to identify potential trends rather than definitive differences. All research analyses were conducted using SPSS version 27.

## 3 Results

This section presents the results of the study. First, the study sample's demographic features were described, then the relationships between those traits and the study's essential variables were examined. Subsequently, the association among the main study variables is reported.

### 3.1 Demographic Information

Out of 35 invited HIS users, 32 completed the questionnaire. Three participants were excluded for returning questionnaires with more than 20% missing responses or for declining to participate in the study. The majority of participants (*n* = 22, 68.8%) were women. Additionally, most participants aged 25–35 (*n* = 17, 53.1%) held a bachelor's degree (*n* = 28, 87.5%) and were nurses (*n* = 17, 53.1%). Notably, *n* = 20, 62.5% of participants had more than 5 years of experience with the system (Table 2). The 32 participants filled out all relevant variables, and there was no evidence of missing data.

**Table 2:** Demographic characteristics of the staff participating in the study.

Variables	Category	Frequency	Percentage (%)
Gender	Male	10	31.2
	Female	22	68.8
Age	< 25	3	9.4
	25–35	17	53.1
	36–50	10	31.2
	> 50	2	6.3
Marital status	Married	26	81.3
	Unmarried	6	18.8
Job position	Radiology	1	3.1
	Secretary	2	6.2
	Laboratory	3	9.3
	Medical records	4	12.5
	Financial	3	9.3
	Pharmacy	2	6.2
	Nursing	17	53.1
Education degree	Associate	1	3.1
	Bachelor	28	87.5
	Master	3	9.4
	Ph.D.	0	0.0
The daily work duration with HIS (hour(s))	< 1	3	9.4
	1–3	11	34.4
	4–6	8	25.0
	6–8	10	31.3
	> 8	0	0.0
Experience working with the system (year(s))	< 1	2	6.3
	1–3	8	25.0
	3–5	2	6.3
	> 5	20	62.5

### 3.2 Exploratory Analysis of the Participants' Demographic Information Based on the Research Variables

We explored the distribution of research variables across different participant demographic groups (Tables 3, 4, 5). Some tendencies in mean scores were observed across job position groups. Paramedical staff showed comparatively higher mean scores for both “user satisfaction” (mean difference = −0.42, 95% CI [−0.82, −0.02]) and “system utilization” (mean difference = −0.25, 95% CI [−0.46, −0.05]) compared to clinicians (Table 3).

**Table 3:** Independent‐sample *t*‐test comparing demographic factor groups in research variables.

Variables	Demographic groups	*N*	Mean	SD	Mean difference	95% CI	df	*t*	*p*	Cohen's *d*
Gender										
Familiarity with IT	Female	22	3.52	0.70	−0.40	−0.97, 0.16	30	−1.44	0.16	−0.54
	Male	10	3.93	0.81						
Training and supporting	Female	22	3.05	0.54	0.05	−4.31, 0.53	30	0.21	0.83	0.08
	Male	10	3.00	0.76						
System utilization	Female	22	3.25	0.34	−0.09	−0.33, 0.14	30	−0.77	0.44	−0.29
	Male	10	3.34	0.19						
Matching the system with users' needs	Female	22	3.17	0.55	−0.36	−0.76, 0.26	30	−1.90	0.06	−0.72
	Male	10	3.54	0.36						
User satisfaction	Female	22	3.75	0.60	−0.23	−0.68, 0.21	30	−1.07	0.29	−0.41
	Male	10	3.99	0.52						
Age categories										
Familiarity with IT	Young adult (≤ 35 years)	20	4.04	0.43	1.04	0.63, 1.46	30	5.19	**< 0.001**	1.89
	Middle adult (> 35 years)	12	2.99	0.71						
Training and supporting	Young adult (≤ 35 years)	20	3.06	0.52	0.08	−0.37, 0.55	30	0.39	0.69	0.14
	Middle adult (> 35 years)	12	2.97	0.75						
System utilization	Young adult (≤ 35 years)	20	3.30	0.25	0.06	−0.16, 0.29	30	0.60	0.55	0.21
	Middle adult (> 35 years)	12	3.23	0.38						
Matching the system with users' needs	Young adult (≤ 35 years)	20	3.24	0.48	−0.12	−0.52, 0.27	30	−0.64	0.522	−0.23
	Middle adult (> 35 years)	12	3.37	0.60						
User satisfaction	Young adult (≤ 35 years)	20	3.82	0.58	−0.01	−0.45, 0.42	30	−0.07	0.94	−0.02
	Middle adult (> 35 years)	12	3.84	0.60						
Job Position										
Familiarity with IT	Clinician	17	3.50	0.92	−0.30	−0.82, 0.22	24.3*	−1.19	0.24	−0.40
	Paramedical	15	3.81	0.46						
Training and supporting	Clinician	17	3.03	0.56	0.008	−0.44, 0.45	30	0.03	0.96	0.01
	Paramedical	15	3.03	0.67						
System utilization	Clinician	17	3.16	0.26	−0.25	−0.46, −0.05	30	−2.60	**0.014**	−0.92
	Paramedical	15	3.42	0.30						
Matching the system with users' needs	Clinician	17	3.17	0.59	−0.24	−0.61, 0.12	28.6^a^	−1.37	0.18	−0.47
	Paramedical	15	3.42	0.41						
User satisfaction	Clinician	17	3.63	0.58	−0.42	−0.82, −0.02	30	−2.17	**0.037**	−0.77
	Paramedical	15	4.05	0.50						
Work Experience										
Familiarity with IT	≤ 5 years	12	3.98	0.48	0.53	0.06, 1.00	30.00^a^	2.30	**0.028**	0.74
	> 5 years	20	3.45	0.81						
Training and supporting	≤ 5 years	12	3.13	0.47	0.26	−0.30, 0.61	30	0.68	0.49	0.25
	> 5 years	20	2.97	0.68						
System utilization	≤ 5 years	12	3.38	0.28	0.15	−0.06, 0.38	30	1.44	0.15	0.52
	> 5 years	20	3.22	0.30						
Matching the system with users' needs	< 5 years	12	3.43	0.54	0.22	−0.16, 0.61	30	1.16	0.25	0.42
	> 5 years	20	3.20	0.51						
User satisfaction	≤ 5 years	12	4.00	0.53	0.26	−0.16, 0.69	30	1.26	0.21	0.46
	> 5 years	20	3.73	0.59						

^a^ The Levene's test indicates that the variance is not equal between the two groups. Bolded values indicate exploratory patterns of potential differences in the mean scores of the research variables across demographic groups.

**Table 4:** Exact Mann–Whitney test for comparing education level groups in the research variables.

Variables	Education levels	*N*	Median	Interquartile range/real data^a^	U	Z	*p*	*r* = (*z*/√*N*)
Familiarity with IT	Undergraduate	29	3.75	0.97	25.00	−1.19	0.25	−0.21
	Postgraduate	3	4.55	4.65, 3.30, 4.55				
Training and supporting	Undergraduate	29	3.00	0.92	40.00	−0.22	0.84	−0.03
	Postgraduate	3	3.13	1.75, 3.63, 3.13				
System utilization	Undergraduate	29	3.30	0.30	42.00	−0.09	0.94	−0.01
	Postgraduate	3	3.15	2.95, 4.13, 3.15				
Matching the system with users' needs	Undergraduate	29	3.45	0.46	39.00	−0.29	0.80	−0.05
	Postgraduate	3	3.18	2.82, 3.45, 3.18				
User satisfaction	Undergraduate	29	3.82	0.44	38.00	−0.35	0.74	−0.06
	Postgraduate	3	3.65	3.18, 4.82, 3.65				

^a^ Since the frequency of the postgraduate subgroup is *n* = 3, the interquartile range (IQR) becomes unreliable or even undefined in a meaningful sense; therefore, the raw data were reported.

**Table 5:** Kruskal–Wallis test for comparing the daily working time duration in the research variables.

Variables	Daily work duration groups (hours)	*N*	Median	Interquartile range/real data^a^	Mean rank	H	df	*p*	Eta‐squared = H/(*N*−1)
Familiarity with IT	< 1	3	2.60	2.55, 3.45, 2.60	7.00	3.42	3	0.33	0.11
	1–3	11	3.95	1.65	17.45				
	4–6	8	3.82	0.63	17.13				
	6–8	10	3.92	0.97	17.80				
Training and supporting	< 1	3	2.88	1.88, 3.33, 2.88	11.67	1.69	3	0.63	0.05
	1–3	11	3.29	0.92	17.68				
	4–6	8	3.33	1.06	18.69				
	6–8	10	2.89	0.97	14.90				
System utilization	< 1	3	2.90	2.65, 3.15, 2.90	5.00	6.87	3	0.07	0.22
	1–3	11	3.20	0.25	14.73				
	4–6	8	3.40	0.22	20.31				
	6–8	10	3.41	0.66	18.85				
Matching the system with users' needs	< 1	3	2.64	2.45, 2.64, 2.73	4.33	8.91	3	**0.030**	0.28
	1–3	11	3.45	0.91	18.14				
	4–6	8	3.63	0.55	22.00				
	6–8	10	3.18	0.75	13.95				
User satisfaction	< 1	3	3.50	2.59, 3.76, 3.50	7.33	4.77	3	0.18	0.15
	1–3	11	3.82	0.38	16.36				
	4–6	8	4.00	0.32	21.00				
	6–8	10	3.77	1.29	15.80				

^a^ Since the frequency of the postgraduate subgroup is *n* = 3, the interquartile range (IQR) becomes unreliable or even undefined in a meaningful sense; therefore, the raw data were reported. Bolded values indicate exploratory differences in the distribution of research variable scores across daily HIS use duration groups.

Patterns in “familiarity with IT” across age groups (mean difference = 1.04, 95% CI [0.63, 1.46]) and work experience groups (mean difference = 0.53, 95% CI [0.06, 1.00]) were also observed (Table 3). The younger adult group and hospital staff with fewer than 5 years of experience working with HIS showed a higher mean score in the “familiarity with IT” variable.

There was no tendency between research variables based on education levels (Table 4). Exploratory subgroup comparisons suggested potential differences in the mean score of “matching the system with users' needs” across categories of daily working time duration (*p* = 0.030, Table 5). Participants with moderate daily usage of the system (e.g., 4–6 h) tended to report higher scores (mean rank = 22.00, median = 3.63, IQR = 0.55) compared to those with minimal usage. However, due to limited and uneven subgroup sizes, these findings should not be interpreted as confirmatory evidence.

While exploratory analyses indicated statistical differences between these background groups, these findings should be interpreted with caution due to the small sample size and heterogeneity of our sample.

### 3.3 Descriptive and Correlational Analysis of Study Variables

Among the study variables, the user's satisfaction has the highest score (mean = 3.83, SD = 0.58), and the “training and supporting” variable has the lowest (mean = 3.03, SD = 0.61) (Table 6).

**Table 6:** Descriptive analysis of research variables.

Research variables	*N*	Mean	SD
Familiarity with IT	32	3.65	0.75
Training and supporting	32	3.03	0.61
System utilization	32	3.28	0.30
Matching the system with users' needs	32	3.29	0.52
User satisfaction	32	3.83	0.58

Based on our findings (Table 7), “user satisfaction” has a statistically significant and strong correlation with “system utilization” (*r* = 0.830, *p* < 0.001) and “matching the system with users' needs” (*r* = 0.626, *p* < 0.001). Furthermore, it has a moderate and significant correlation with the “training and supporting” variable (*r* = 0.597, *p* < 0.001). Another strong, statistically significant correlation is between “matching the system with users' needs” and “system utilization” (*r* = 0.713, *p* < 0.001). According to the positive correlation coefficient, these relationships are direct.

**Table 7:** The correlations between the research variables (correlation coefficient, *p*‐value).

Variables	Familiarity with IT	Training and supporting	System utilization	Matching the system with users' needs	User satisfaction
Familiarity with IT	1				
Training and supporting	0.128^b^, (0.486)	1			
	95% CI [−0.32, 0.52]				
System utilization	0.088^b^, (0.632)	0.474^a^c, (0.061)	1		
	95% CI [−0.25, 0.43]	95% CI [0.15, 0.69]			
Matching the system with users' needs	0.044^b^, (0.812)	0.311^b^, (0.084)	**0.713** a, **(*p*** < **0.001)**	1	
	95% CI [−0.31, 0.42]	95% CI [−0.004, 0.57]	95% CI [0.46, 0.85]		
User satisfaction	0.073^b^, (0.692)	**0.597** a, **(*p*** < **0.001)**	**0.830** a, **(*p*** < **0.001)**	**0.626** a, **(*p*** < **0.001)**	1
	95% CI [−0.23, 0.37]	95% CI [0.33, 0.80]	95% CI [0.67, 0.91]	95% CI [0.31, 0.81]	

^a^ The correlation is significant at the 0.01 level (two‐tailed).

^b^ The correlation is significant at the 0.05 level (two‐tailed). Bolded values indicate statistically significant correlations between research variables.

Based on a one‐sample *t*‐test (assumption: *µ* = 3) for the research variables (Table 8), there are statistically significant differences in the mean scores for “familiarity with IT” (mean difference = 0.65, 95% CI [0.38, 0.92], *p* < 0.001), “matching system with users' needs” (mean difference = 0.29, 95% CI [0.10, 0.48], *p* = 0.004), “system utilization” (mean difference = 0.28, 95% CI [0.17, 0.39], *p* < 0.001), and “user satisfaction” (mean difference = 0.83, 95% CI [0.62, 1.04], *p* < 0.001) at the neutral value of 3. Because the mean scores of these variables are greater than 3, it can be concluded that, from the participants' viewpoint, the hospital staff has a high degree of familiarity with IT, the HIS system aligns well with its users' requirements, the level of HIS utilization in this research was higher than the average, and the satisfaction with the HIS in this hospital is relatively high.

**Table 8:** One‐sample *t*‐test of the research variables.

Variables	*N*	Mean	SD	Mean difference	95% CI	df	*t*	*p*‐value	Cohen's *d*
Familiarity with IT	32	3.65	0.75	0.65	0.38, 0.92	31	4.91	**< 0.001**	0.86
Training and supporting	32	3.03	0.61	0.03	−0.18, 0.25	31	0.32	0.74	0.05
Matching the system with users' needs	32	3.29	0.52	0.29	0.10, 0.48	31	3.14	0.004	0.55
System utilization	32	3.28	3.06	0.28	0.17, 0.39	31	5.20	**< 0.001**	0.92
User satisfaction	32	3.83	0.58	0.83	0.62, 1.04	31	8.10	**< 0.001**	1.43

**Figure 2:**
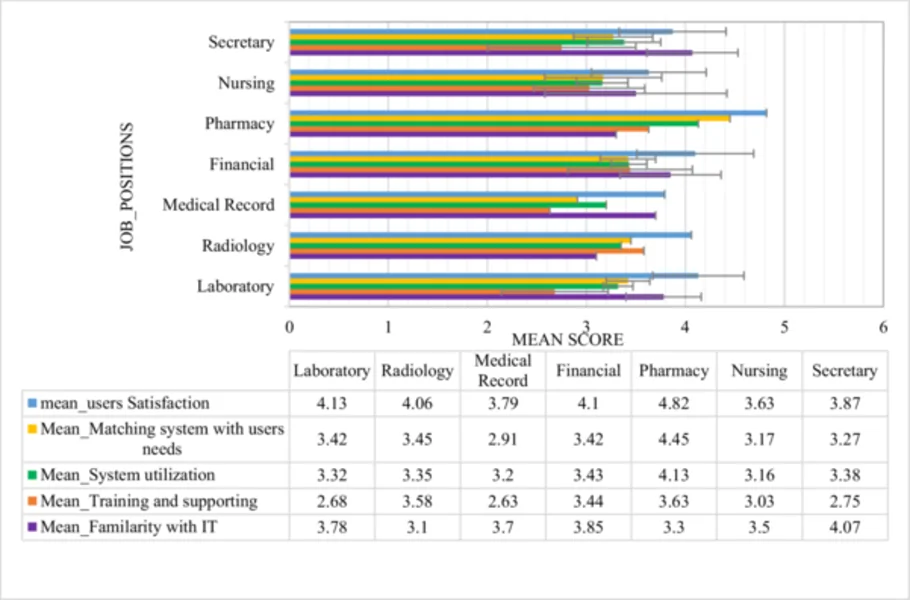
Research variables and participants' job positions.

According to Figure 2, among participants in this study across different occupations, pharmacy technicians used the system most frequently (score = 4.13), while nurses used it least frequently (mean = 3.16, SD = 0.26). Additionally, pharmacy technicians reported the highest level of satisfaction with this system (score = 4.82), while nurses reported the lowest level (mean = 3.63, SD = 0.58).

## 4 Discussion

Despite the motivations for implementing HISs in Iran, evidence shows that these systems do not meet user needs [39]. This study examined the relationships between familiarity with IT, training and supporting, matching the system with users' needs, user satisfaction, and system utilization within the context of a HIS in a tertiary hospital in Iran. By integrating concepts from TAM and UTAUT and explicitly considering user attitude in a mandatory‐use environment, the study aimed to provide a broader understanding of HIS utilization in LMIC settings.

The variable “matching the system with users' needs” has a significant, positive relationship with both “users' satisfaction” and “system utilization” (Table 7). This finding highlights the importance of perceived usefulness and task–technology alignment, which are core concepts in technology acceptance theories such as TAM [25]. Users who perceived that the HIS supported their workflow, tasks, and operational needs were more likely to report satisfaction and greater system utilization [40]. In mandatory‐use environments, such as public hospitals in Iran, system utilization may not depend solely on users' behavioral intention, because employees are often required to use the system regardless of their personal preferences. Under these conditions, the extent to which the system aligns with users' practical needs may become a more influential determinant of effective utilization than voluntary motivation alone [41]. This interpretation is consistent with previous studies that emphasized the importance of system compatibility, workflow integration, and perceived usefulness in promoting health information system adoption and continued use [42, 43]. However, our findings contrast with studies that reported weaker relationships between system fit and user outcomes [44, 45], which may reflect differences in organizational context, implementation maturity, or mandatory‐use policies.

The significant relationship between user satisfaction and system utilization (Table 7) also supports the theoretical assumptions of both TAM and UTAUT [25, 26], in which positive user perceptions contribute to continued technology use. Satisfaction appears to function not only as an outcome of successful system interaction but also as a reinforcing factor that promotes repeated and sustained system utilization [46, 47]. In practical settings, repeated use may increase users' familiarity with system functionalities, improve task efficiency, and reduce uncertainty during workflow execution. Over time, this may strengthen users' confidence and contribute to more favorable attitudes toward the HIS [8, 48]. Similar findings have been reported in previous studies examining electronic health record usability and health information system acceptance [43, 49, 50]. Therefore, improving user satisfaction may play an important role in promoting long‐term HIS utilization and implementation success.

Another important finding concerns the role of training and support. Although training and support showed a significant correlation with user satisfaction, their relationship with system utilization was positive but not statistically significant (Table 7). From the perspective of UTAUT [26], training and organizational support can be interpreted as facilitating conditions that help users engage effectively with technology. However, the absence of a strong direct association with utilization in this study may indicate that the existing training approaches were insufficiently aligned with users' daily workflow requirements or were not delivered at appropriate times during system implementation [51]. In mandatory‐use settings, users may continue using the HIS regardless of training quality because system use is institutionally required. Consequently, training may influence utilization indirectly through improving users' comfort, confidence, and satisfaction rather than directly affecting whether the system is used [52, 53]. This interpretation is supported by studies emphasizing that the effectiveness of HIS training depends on relevance, usability‐oriented instruction, and organizational integration rather than the mere availability of training programs [51, 54]. Therefore, hospitals may benefit from redesigning training programs to focus on practical workflow scenarios, continuous support, and user‐centered learning strategies.

Prior research [55, 56, 57, 58, 59] has shown that demographic variables (e.g., age or gender) are associated with differences in system use, usability perception, and training needs. From a system design and implementation perspective, these factors can help identify user groups that may require targeted training, interface adjustments, or workflow adaptations.

The findings related to familiarity with IT and demographic characteristics provide additional insight into technology adoption behavior (Table 3). Younger participants and users with fewer years of work experience demonstrated higher familiarity with IT, which is consistent with previous literature suggesting that age and prior exposure to technology influence digital competency [60, 61]. Nevertheless, familiarity with IT did not show a significant relationship with system utilization (Table 7). This finding may reflect the long‐term and mandatory use of the HIS in the studied hospital. Over time, repetitive exposure to the system may reduce differences between users with varying technological backgrounds, allowing even users with limited IT skills to achieve functional system utilization. In this context, institutional routines and workflow dependence may compensate for lower digital literacy [62, 63, 64, 65, 66]. This finding suggests that technical skill alone may not adequately explain HIS utilization in mature mandatory‐use environments and that organizational and contextual factors should also be considered.

The exploratory analysis reveals different levels of user satisfaction based on job position (Table 3). This is consistent with the results of the study by Abbasi et al. [55] but contradicts those of previous studies, such as Ebnehoseini et al. [56], which reported a significant relationship between satisfaction and education level, and Sulley et al. [59], which demonstrated a significant relationship between physician age and satisfaction with hospital information systems. This may be due to the growing attention to user characteristics in the design of information systems [67, 68]. Therefore, we can assume that the effect of demographic factors on user satisfaction decreases over time; however, this requires further examination in future research.

A limitation of this study is the absence of physicians and managerial staff, who play a central role in clinical decision‐making and system evaluation. could have introduced bias in the selection of clinical and administrative staff. Therefore, the findings primarily reflect the perspectives of operational users involved in daily system use. While these users provide important insights into usability and workflow integration, the results may not fully capture requirements related to clinical decision‐making or strategic system development. Future studies should include physicians and managers to enable a more comprehensive evaluation. Although the sample size (*n* = 32) was appropriate for the total population of active HIS users in the studied hospital, it remains relatively small. As a result, the findings should be interpreted with caution when considering generalizability to other hospitals or larger user populations. The small sample may reduce statistical power and limit the ability to detect weaker associations [69]. More research with a larger sample size is suggested to validate and expand upon these results. The study conclusions may also not apply to other hospitals that use different vendors' HIS systems.

## 5 Conclusion

This research aims to investigate a system's compatibility with its users' needs and identify the factors that influence the system's use in achieving implementation goals. A researcher‐made questionnaire based on well‐known models concerning technology acceptance in a healthcare context was developed. We developed this questionnaire to consider both mental and practical aspects of users' behavior in adopting the HIS system. Our findings confirm the central role of responding to users' needs and ensuring user satisfaction in the utilization of the HIS system. However, regarding the significant correlation between user satisfaction and training and support, it can be concluded that training and support can indirectly influence the HIS system utilization in the studied hospital.

Based on the study results, further research is recommended to investigate the relationship between the increasing demands on the HIS changes and the rejection of using unsuitable ones due to growing experience with them, as well as exploring the declining effect of demographic factors on users' satisfaction over time. Regarding the nonsignificant correlation between training and support and system utilization, we propose to the studied hospital managers that it is essential to reorganize and evaluate the training program at the hospital. We also suggested that combining behavioral and cognitive models related to healthcare environments in future research can help understand other dimensions of system acceptance and exploitation.

## Author Contributions

**Fatemeh Aghilinasab:** writing – original draft, methodology, visualization, validation, writing – review and editing, formal analysis, investigation, software. **Mozhgan Tanhapour:** investigation, writing – original draft, writing – review and editing, formal analysis, validation. **Mohamad Jebraeily:** investigation, writing – review and editing, methodology. **Bahlol Rahimi:** conceptualization, investigation, writing – review and editing, methodology, validation, supervision, project administration, software.

## Funding

The authors have nothing to report.

## Disclosure

The Fatemeh Aghilinasab affirms that this manuscript is an honest, accurate, and transparent account of the study being reported; that no important aspects of the study have been omitted; and that any discrepancies from the study as planned (and, if relevant, registered) have been explained.

## Ethics Statement

This study was part, approved by the Ethics Committee of Urmia University of Medical Sciences (IR.UMSU.REC.1398.104). All procedures were performed under the guidelines and the rules of the Urmia University of Medical Sciences Ethics Committee. The participants signed an informed consent form before participating in the study.

## Conflicts of Interest

The authors declare no conflicts of interest.

## Data Availability

The data that support the findings of this study are available in the Supporting Material of this article.

## References

[1] E. Ermetov , D. Yakhshiboyeva , V. Maxsudov , and R. J. S. Yakhshiboyev , “Innovation,” Importance of Information Technologies in Preserving Health 2, no. A4 (2023): 92–95.

[2] A. Winter , E. Ammenwerth , R. Haux , M. Marschollek , B. Steiner , and F. Jahn , Health Information Systems: Technological and Management Perspectives (Springer Nature, 2023). 38598651

[3] M. F. Collen and R. A. Miller , “The Early History of Hospital Information Systems for Inpatient Care in the United States.” The History of Medical Informatics in the United States (Springer, 2015), 339–383.

[4] A. Hussain , M. Zhiqiang , M. Li , et al., “The Mediating Effects of Perceived Usefulness and Perceived Ease of Use on Nurses' Intentions to Adopt Advanced Technology,” BMC Nursing 24, no. 1 (2025): 33. 39789568 10.1186/s12912-024-02648-8 PMC11716174

[5] M. Khalifa and O. Alswailem , “Hospital Information Systems (HIS) Acceptance and Satisfaction: A Case Study of a Tertiary Care Hospital,” Procedia Computer Science 63 (2015): 198–204.

[6] F. Sadoughi , K. Kimiafar , M. Ahmadi , and M. T. Shakeri , “Determining of Factors Influencing the Success and Failure of Hospital Information System and Their Evaluation Methods: A Systematic Review,” Iranian Red Crescent Medical Journal 15, no. 12 (1970): e11716. 10.5812/ircmj.11716 PMC3955501 24693386

[7] N. Hendrix , A. Bazemore , A. J. Holmgren , et al., “Variation in Family Physicians' Experiences Across Different Electronic Health Record Platforms: A Descriptive Study,” Journal of General Internal Medicine 38, no. 13 (2023): 2980–2987. 36952084 10.1007/s11606-023-08169-5 PMC10035476

[8] A. J. Holmgren , N. Hendrix , N. Maisel , et al., “Electronic Health Record Usability, Satisfaction, and Burnout for Family Physicians,” JAMA Network Open 7, no. 8 (2024): e2426956‐e. 39207759 10.1001/jamanetworkopen.2024.26956 PMC11362862

[9] M. Jansson , J. Liisanantti , T. Ala‐Kokko , and J. Reponen , “The Negative Impact of Interface Design, Customizability, Inefficiency, Malfunctions, and Information Retrieval on User Experience: A National Usability Survey of ICU Clinical Information Systems in Finland,” International Journal of Medical Informatics 159 (2022): 104680. 34990942 10.1016/j.ijmedinf.2021.104680

[10] D. Schwappach , W. Hautz , G. Krummrey , Y. Pfeiffer , and R. M. Ratwani , “EMR Usability and Patient Safety: A National Survey of Physicians,” npj Digital Medicine 8, no. 1 (2025): 282. 40374898 10.1038/s41746-025-01657-4 PMC12081653

[11] H. Ahmadi , M. Nilashi , L. Shahmoradi , and O. Ibrahim , “Hospital Information System Adoption: Expert Perspectives on an Adoption Framework for Malaysian Public Hospitals,” Computers in Human Behavior 67 (2017): 161–189.

[12] H. Ahmadi , M. Nilashi , O. Ibrahim , et al., “Exploring Potential Factors in Total Hospital Information System Adoption,” Journal of Soft Computing Decision Support Systems 2, no. 1 (2015): 52–59.

[13] N. I. Ismail , N. H. Abdullah , and A. Shamsuddin , “Adoption of Hospital Information System (HIS) in Malaysian Public Hospitals,” Procedia ‐ Social and Behavioral Sciences 172 (2015): 336–343.

[14] H. Khorasani , S. Damiri , S. Abbasi , A. Fathabadi , M. Dehghani , and M. Sadeghi , “Hospital Information Systems Evaluation Criteria: A Scoping Review,” BMC Health Services Research 26 (2026): 184. 41501780 10.1186/s12913-025-13972-3 PMC12882420

[15] A. R. Ahlan and B. I. Ahmad , “User Acceptance of Health Information Technology (HIT) in Developing Countries: A Conceptual Model,” Procedia Technology 16 (2014): 1287–1296.

[16] P. W. Handayani , A. N. Hidayanto , A. A. Pinem , P. I. Sandhyaduhita , and I. Budi , “Hospital Information System User Acceptance Factors: User Group Perspectives,” Informatics for Health and Social Care 43, no. 1 (2018): 84–107. 28140717 10.1080/17538157.2016.1269109

[17] B. Rahimi , R. Safdari , and M. Jebraeily , “Development of Hospital Information Systems: User Participation and Factors Affecting It,” Acta Informatica Medica 22, no. 6 (2014): 398. 25684849 10.5455/aim.2014.22.398-401 PMC4315630

[18] P. Venugopal , S. Jinka , and S. Priya , “User Acceptance of Electronic Health Records: Cross Validation of UTAUT Model,” Global Management Review 10, no. 3 (2016): 42–54.

[19] N. Zakaria and S. A. Mohd Yusof , “Understanding Technology and People Issues in Hospital Information System (HIS) Adoption: Case Study of a Tertiary Hospital in Malaysia,” Journal of Infection and Public Health 9, no. 6 (2016): 774–780. 27686258 10.1016/j.jiph.2016.08.017

[20] S. Barzegari , M. Ghazisaeedi , F. Askarian , A. Jesmi , H. Gandomani , and A. Hasani , “Hospital Information System Acceptance Among the Educational Hospitals,” Journal of Nursing and Midwifery Sciences 7, no. 3 (2020): 186–193.

[21] A. Ismail , A. T. Jamil , A. F. A. Rahman , J. M. A. Bakar , N. M. Saad , and H. Saadi , “The Implementation of Hospital Information System (HIS) in Tertiary Hospitals in Malaysia: A Qualitative Study,” Malaysian Journal of Public Health Medicine 10, no. 2 (2010): 16–24.

[22] F. Kitsios , M. Kamariotou , V. Manthou , and A. Batsara , “Editors. Hospital Information Systems: Measuring End‐User Satisfaction.” Information Systems: 17th European, Mediterranean, and Middle Eastern Conference, EMCIS 2020 (November 25–26: Springer, 2020, Proceedings), 2020, 17.

[23] K. B. Asha , B. Reshmi , and G. Skaria , “IJCM_424A: User Experience and Attitude of End Users Towards Laboratory Information System at a Tertiary Care Hospital,” supplement, Indian Journal of Community Medicine 49, no. Suppl 1 (2024): S121.

[24] S. Y. Y. Tun and S. Madanian , “Clinical Information System (CIS) Implementation in Developing Countries: Requirements, Success Factors, and Recommendations,” Journal of the American Medical Informatics Association 30, no. 4 (2023): 761–774. 36749093 10.1093/jamia/ocad011 PMC10018272

[25] F. D. Davis , “Technology Acceptance Model: TAM,” Information Seeking Behavior Technology Adoption 205, no. 219 (1989): 5.

[26] A. M. Momani , “The Unified Theory of Acceptance and Use of Technology: A New Approach in Technology Acceptance,” International Journal of Sociotechnology and Knowledge Development 12, no. 3 (2020): 79–98.

[27] A. Chang , “UTAUT and UTAUT 2: A Review and Agenda for Future Research,” Winners 13, no. 2 (2012): 10–114.

[28] R. J. Holden and B.‐T. Karsh , “The Technology Acceptance Model: Its Past and Its Future in Health Care,” Journal of Biomedical Informatics 43, no. 1 (2010): 159–172. 19615467 10.1016/j.jbi.2009.07.002 PMC2814963

[29] B. Rahimi , A. Moberg , T. Timpka , and V. Vimarlund , “Editors. Implementing an Integrated Computerized Patient Record System: Towards an Evidence‐Based Information System Implementation Practice in Healthcare,” AMIA Annual Symposium Proceedings 2008 (2008): 616–620. 18999062 PMC2655989

[30] C. Or , “The Role of Attitude in the Unified Theory of Acceptance and Use of Technology: A Meta‐Analytic Structural Equation Modelling Study,” International Journal of Technology in Education and Science 7, no. 4 (2023): 552–570.

[31] B. Rahimi , H. Nadri , H. Lotfnezhad Afshar , and T. Timpka , “A Systematic Review of the Technology Acceptance Model in Health Informatics,” Applied Clinical Informatics 09, no. 03 (2018): 604–634. 10.1055/s-0038-1668091 PMC6094026 30112741

[32] P. W. Handayani , A. N. Hidayanto , A. A. Pinem , I. C. Hapsari , P. I. Sandhyaduhita , and I. Budi , “Acceptance Model of a Hospital Information System,” International Journal of Medical Informatics 99 (2017): 11–28. 28118918 10.1016/j.ijmedinf.2016.12.004

[33] A. A. Rezigalla , “Observational Study Designs: Synopsis for Selecting an Appropriate Study Design,” Cureus 12, no. 1 (2020): e6692. 31988824 10.7759/cureus.6692 PMC6970097

[34] F.‐Y. Pai and K.‐I. Huang , “Applying the Technology Acceptance Model to the Introduction of Healthcare Information Systems,” Technological Forecasting and Social Change 78, no. 4 (2011): 650–660.

[35] K. S. Taber , “The Use of Cronbach's Alpha When Developing and Reporting Research Instruments in Science Education,” Research in Science Education 48, no. 6 (2018): 1273–1296.

[36] W. G. Cochran , Sampling Techniques (John Wiley & Sons, 1977). 3rd ed ed.

[37] M. Elfil and A. Negida , “Sampling Methods in Clinical Research; An Educational Review,” Emergency (Tehran, Iran) 5, no. 1 (2017): e52. 28286859 PMC5325924

[38] D. G. Altman , Practical Statistics for Medical Research (Chapman and Hall/CRC, 1990).

[39] J. Alipour , A. Karimi , S. Ebrahimi , F. Ansari , and Y. Mehdipour , “Success or Failure of Hospital Information Systems of Public Hospitals Affiliated With Zahedan University of Medical Sciences: A Cross Sectional Study in the Southeast of Iran,” International Journal of Medical Informatics 108 (2017): 49–54. 29132631 10.1016/j.ijmedinf.2017.10.005

[40] F. Alsohime , M.‐H. Temsah , A. Al‐Eyadhy , et al., “Satisfaction and Perceived Usefulness With Newly‐Implemented Electronic Health Records System Among Pediatricians at a University Hospital,” Computer Methods and Programs in Biomedicine 169 (2019): 51–57. 30638591 10.1016/j.cmpb.2018.12.026

[41] A. Bhattacherjee , C. J. Davis , A. J. Connolly , and N. Hikmet , “User Response to Mandatory IT Use: A Coping Theory Perspective,” European Journal of Information Systems 27, no. 4 (2018): 395–414.

[42] H. Ayatollahi , M. Langarizadeh , and H. Chenani , “Confirmation of Expectations and Satisfaction With Hospital Information Systems: A Nursing Perspective,” Healthcare Informatics Research 22, no. 4 (2016): 326–332. 27895965 10.4258/hir.2016.22.4.326 PMC5116545

[43] L. R. Kalankesh , Z. Nasiry , R. Fein , and S. Damanabi , “Factors Influencing User Satisfaction With Information Systems: A Systematic Review,” Galen Medical Journal 9 (2020): e1686. 34466567 10.31661/gmj.v9i0.1686 PMC8343607

[44] N. K. Mensah , G. Adzakpah , J. Kissi , et al., “Health Professionals' Perceptions of Electronic Health Records System: A Mixed Method Study in Ghana,” BMC Medical Informatics and Decision Making 24, no. 1 (2024): 254. 39285423 10.1186/s12911-024-02672-3 PMC11403855

[45] B. Tilahun and F. Fritz , “Comprehensive Evaluation of Electronic Medical Record System Use and User Satisfaction at Five Low‐Resource Setting Hospitals in Ethiopia,” JMIR Medical Informatics 3, no. 2 (2015): e4106. 10.2196/medinform.4106 PMC4460264 26007237

[46] B. Hadji and P. Degoulet , “Information System End‐User Satisfaction and Continuance Intention: A Unified Modeling Approach,” Journal of Biomedical Informatics 61 (2016): 185–193. 27033175 10.1016/j.jbi.2016.03.021

[47] B. Hadji , I. Dupuis , L. Leneveut , D. Heudes , J.‐F. Wagner , and P. Degoulet , “Determinants of Continuance Intention in a Post‐Adoption Satisfaction Evaluation of a Clinical Information System.” e‐Health–For Continuity of Care (IOS Press, 2014), 990–994. 25160336

[48] R.‐F. Chen and J.‐L. Hsiao , “Health Professionals' Perspectives on Electronic Medical Record Infusion and Individual Performance: Model Development and Questionnaire Survey Study,” JMIR Medical Informatics 9, no. 11 (2021): e32180. 34851297 10.2196/32180 PMC8672292

[49] E. Agyemang , A. B. Adu‐Gyamfi , E. K. Achampong , and K. Esia‐Donkoh , “Assessing the Interdependency Among Effectiveness, Satisfaction and Efficient Use of the Lightwave Health Information Management System (LHIMS) by Health Professionals in Ghana,” BMC Health Services Research 24, no. 1 (2024): 1418. 39550605 10.1186/s12913-024-11883-3 PMC11568627

[50] S. Wondwossen , M. A. Asemahagn , and H. A. Guadie , “Assessing Electronic Medical Record Usage in Private Healthcare Facilities of Amhara Region, Ethiopia,” Scientific Reports 15, no. 1 (2025): 38311. 41184305 10.1038/s41598-025-22186-w PMC12583491

[51] T. Jeyakumar , S. McClure , M. Lowe , et al., “An Education Framework for Effective Implementation of a Health Information System: Scoping Review,” Journal of Medical Internet Research 23, no. 2 (2021): e24691. 33625370 10.2196/24691 PMC7946593

[52] S. Ajami and M.‐B. Zjjitse , “Training and Its Impact on Hospital Information System (HIS) Success,” Information Technology & Software Engineering 2, no. 05 (2012): 1000112.

[53] K. E. Robinson and J. A. Kersey , “Novel Electronic Health Record (EHR) Education Intervention in Large Healthcare Organization Improves Quality, Efficiency, Time, and Impact on Burnout,” Medicine 97, no. 38 (2018): e12319. 30235684 10.1097/MD.0000000000012319 PMC6160120

[54] J. Campione and H. Liu , “Perceptions of Hospital Electronic Health Record (EHR) Training, Support, and Patient Safety by Staff Position and Tenure,” BMC Health Services Research 24, no. 1 (2024): 955. 39164672 10.1186/s12913-024-11322-3 PMC11337607

[55] R. Abbasi , M. Sadeqi Jabali , R. Khajouei , and H. Tadayon , “Investigating the Satisfaction Level of Physicians in Regards to Implementing Medical Picture Archiving and Communication System (PACS),” BMC Medical Informatics and Decision Making 20, no. 1 (2020): 180. 32758220 10.1186/s12911-020-01203-0 PMC7405331

[56] Z. Ebnehoseini , M. Jangi , M. Tara , and H. Tabesh , “Investigation the Success Rate of Hospital Information System (HIS): Development of a Questionnaire and Case Study,” Journal of Healthcare Quality Research 36, no. 2 (2021): 103–112. 33495115 10.1016/j.jhqr.2020.03.010

[57] F. R. Jeddi , E. Nabovati , R. Bigham , and R. Khajouei , “Usability Evaluation of a Comprehensive National Health Information System: Relationship of Quality Components to Users' Characteristics,” International Journal of Medical Informatics 133 (2020): 104026. 31733603 10.1016/j.ijmedinf.2019.104026

[58] J. I. Nicholars and M. Kantaris , “Influence of Demographic Characteristics on Health Workers' Acceptance of Biometric‐Controlled Health Informatics Systems: Evidence From Selected Regional Referral Public Hospitals in Uganda,” Journal of Health Informatics in Africa 13, no. 1 (2026): 66–77.

[59] S. Sulley and M. Ndanga , “Influence of EHR System Type and Physician Demographics on Satisfaction Levels in Health Information Technology,” Discover Public Health 22, no. 1 (2025): 119.

[60] T. Aleti , B. Figueiredo , M. Reid , D. M. Martin , J. Sheahan , and L. Hjorth , “Older Adults' Digital Competency, Digital Risk Perceptions and Frequency of Everyday Digital Engagement,” Information Technology & People 38, no. 8 (2025): 97–118.

[61] J. Deane , M. Bradley , and P. J. Clarkson , “Digital Technology Competence and Experience in the UK Population: Who Can Do What.” Ergonomics and Human Factors (Apollo ‐ University of Cambridge Repository, 2020).

[62] K. Alwan , T. A. Ayele , and B. Tilahun , “Knowledge and Utilization of Computers Among Health Professionals in a Developing Country: A Cross‐Sectional Study,” JMIR Human Factors 2, no. 1 (2015): e4184. 10.2196/humanfactors.4184 PMC4797659 27025996

[63] L. Kayser , A. Karnoe , E. Duminski , et al., “Health Professionals' eHealth Literacy and System Experience Before and 3 Months After the Implementation of an Electronic Health Record System: Longitudinal Study,” JMIR Human Factors 9, no. 2 (2022): 29780. 10.2196/29780 PMC9107047 35486414

[64] A. Magnusson and E. Hall Andreasson , Advantages and Limitations of EHR Use in Clinical Workflows: Physicians' Experiences from a Socio‐Technical Perspective (Lund University, 2025).

[65] M. N. Sibiya , O. R. Akinyemi , and O. Oladimeji , “Computer Skills and Electronic Health Records (EHRs) in a State Tertiary Hospital in Southwest Nigeria,” Epidemiologia 4, no. 2 (2023): 137–147. 37218874 10.3390/epidemiologia4020015 PMC10204356

[66] N. Zaman , D. M. Goldberg , S. Kelly , R. S. Russell , and S. L. Drye , “The Relationship Between Nurses' Training and Perceptions of Electronic Documentation Systems,” Nursing Reports 11, no. 1 (2021): 12–27. 34968308 10.3390/nursrep11010002 PMC8608127

[67] B. Fischer , A. Peine , and B. Östlund , “The Importance of User Involvement: A Systematic Review of Involving Older Users in Technology Design,” Gerontologist 60, no. 7 (2020): e513–e523. 31773145 10.1093/geront/gnz163 PMC7491439

[68] S. Johansson , P.‐O. Hedvall , M. Larsdotter , T. P. Larsson , and C. Gustavsson , “Co‐Designing With Extreme Users: A Framework for User Participation in Design Processes,” Scandinavian Journal of Disability Research 25, no. 1 (2023): 418–430.

[69] W. Gan , “Impact of Sample Size and Its Estimation in Medical Research,” AJPM Focus 5 (2025): 100451. 42272553 10.1016/j.focus.2025.100451 PMC13246433

